# Feasibility Study on a Microwave-Based Sensor for Measuring Hydration Level Using Human Skin Models

**DOI:** 10.1371/journal.pone.0153145

**Published:** 2016-04-05

**Authors:** Rico Brendtke, Michael Wiehl, Florian Groeber, Thomas Schwarz, Heike Walles, Jan Hansmann

**Affiliations:** 1 Chair Tissue Engineering and Regenerative Medicine (TERM), University Hospital Wuerzburg, Roentgenring 11, 97070, Wuerzburg, Germany; 2 senetics healthcare group GmbH & Co. KG, Henkestrasse 91, 91052, Erlangen, Germany; 3 Translational Center in oncology Wuerzburg ´Regenerative therapies and musculoskeletal diseases` Wuerzburg, Fraunhofer Institute for Interfacial Engineering and Biotechnology (IGB), Roentgenring 11, 97070, Wuerzburg, Germany; University of Zurich, SWITZERLAND

## Abstract

Tissue dehydration results in three major types of exsiccosis—hyper-, hypo-, or isonatraemia. All three types entail alterations of salt concentrations leading to impaired biochemical processes, and can finally cause severe morbidity. The aim of our study was to demonstrate the feasibility of a microwave-based sensor technology for the non-invasive measurement of the hydration status. Electromagnetic waves at high frequencies interact with molecules, especially water. Hence, if a sample contains free water molecules, this can be detected in a reflected microwave signal. To develop the sensor system, human three-dimensional skin equivalents were instituted as a standardized test platform mimicking reproducible exsiccosis scenarios. Therefore, skin equivalents with a specific hydration and density of matrix components were generated and microwave measurements were performed. Hydration-specific spectra allowed deriving the hydration state of the skin models. A further advantage of the skin equivalents was the characterization of the impact of distinct skin components on the measured signals to investigate mechanisms of signal generation. The results demonstrate the feasibility of a non-invasive microwave-based hydration sensor technology. The sensor bears potential to be integrated in a wearable medical device for personal health monitoring.

## Introduction

In biological systems, water represents the basic building block, a solvent, a reaction medium, and a carrier of nutrients and waste products. Furthermore, it plays an important role in thermoregulation or presents a lubricant and shock absorber [1]. Currently, water-related diseases are a growing global challenge, killing more than 5 million people per year [2]. Thus, hydration is an essential vital parameter [3]. Dehydration decreases the total water volume and results in a reduced central blood volume [4]. This induces thermoregulatory stress, hyper osmolality, and impaired physical conditions [5–8]. In contrast to nutrient deficiencies that take weeks up to years to arise, a body is seriously dehydrated within a few days without water uptake. Tissue dehydration entails three major types of exsiccosis. All of these are related to osmolality alterations due to changes in salt concentrations; mainly sodium and chloride [9]. A tissue that only loses water suffers from hypernatraemia resulting in a high salt concentration. If salt molecules are depleted, hyponatraemia occurs. If a tissue lacks both water and salt molecules, isotonic dehydration is the consequence. Possible loss of water is caused by perspiration, exhaled air, urine, loss through intestine and feces [10]. Severe or chronic dehydration is a strong trigger for the development of acute or chronic diseases. The amount of compensatory water uptake individually depends on the physical activity and the ambient conditions such as temperature or humidity.

To ensure a healthy lifestyle and to prevent morbidity, personal health monitoring (PHM) technologies are employed [11], which are of particular importance to people at higher ages. PHM technologies need to be robust and should record health information non-invasively to facilitate applicability in everyday life and special care [12,13]. Currently, several PHM systems access vital parameters such as blood oxygenation or heart rate through the skin [14,15]. Regarding hydration, the correlation between the water content of the skin surface and the body’s hydration state has already been demonstrated [16]. Technologies to measure the water content in the tissue allowing a more accurate prediction of body hydration are currently not available.

Due to substance-specific interactions between electromagnetic waves and a sample [17,18], microwave-based sensors can be used to measure the water content of the skin non-invasively. Interactions of microwaves with water molecules cause local molecule movements. Thereby, emitted microwave signals are partially absorbed. By detecting the reflected signal intensity within a specific frequency range, the water content can be measured. However, the development of a microwave-based skin hydration sensor is challenging since native skin exhibits a high complexity. Additionally, skin anatomy depends on sites of excision and subject [19]. Regarding sensor characterization and calibration, accessing water content of native skin in order to correlate a sensor signal to a specific hydration state requires time-consuming analyses and tests [20]. Thus, a key criterion for sensor development and characterization are standardized skin samples with defined water content in a sufficient quantity. As an alternative to native skin samples, tissue-engineered skin equivalents of different complexity—from simple epidermal models to multi-layered full-thickness skin tissues—can be generated [21]. These represent standardized model systems due to their capacity to resemble skin anatomy and physiology [22], and are, for instance, used in pharmacological drug development [23]. In contrast to measurements performed *in vivo* or *ex vivo* on excised skin samples, skin equivalents further allow investigating the influence of distinct skin layers on the measurement to fully understand underlying mechanisms of signal generation.

The aim of this study was to investigate whether microwave-based sensor technologies can be instituted as novel diagnostic tools for monitoring relative changes of hydration in biological systems. Therefore, human three-dimensional (3D) skin equivalents were employed as a standardized test platform mimicking reproducible exsiccosis scenarios.

## Material and Methods

Cell culture medium and buffers were purchased from Life Technologies GmbH, Darmstadt, Germany, if not stated otherwise.

Primary cells were isolated from foreskin biopsies from juvenile donors aged between 1 and 3 years under informed consent according to ethical approval granted by the institutional ethics committee of the Julius-Maximilians-University Wuerzburg. The study was approved by the ethics committee (vote 182/10). Additionally, informed and written consent from the next of kin, caretakers, or guardians on behalf of the children was obtained.

### Bioreactor design and plastic compression device

A bioreactor and a plastic compression device were used to produce skin models exhibiting defined and consistent dimensions. The design of the bioreactor was conducted in a computer aided design environment (SolidWorks; Dassault Systèmes SolidWorks Corp.; Waltham, USA). From 3D design files, two-dimensional technical drawings were derived and sent to a fabrication facility (GT Labortechnik, Arnstein, Germany). There, parts of the bioreactor were manufactured from polysulfone (PSU LSG nature).

To diminish model contraction caused by the tensile forces of cells, we plastically compressed the dermal equivalents subsequently to gelation. Therefore, a linear motion engine LinMot^®^ PS01-37x120F-HP-C (LinMot USA Inc., Elkhorn, USA) and an individually-designed stamp from brushed stainless steel were used. The linear motion engine was steered via a tailored user interface based on a SIMATIC programming environment (Siemens AG, Berlin, Germany). Plastic compression was performed allowing a consistent final volume of the dermal equivalents of *V* = 3.6 cm^3^ with a height of *h* = 0.8 cm at a compression velocity of *v* = 1.3x10^-5^ m/s.

### Microwave reflection measurement

RO4350 laminate (Rogers Corporation, Gent, Belgium) was used as substrate material to build a broad-band antenna with an optimal match at the resonance frequency of water molecules at 7.35 GHz. The microwave-emitting patch antenna was developed employing the Sonnet Lite simulation software (Sonnet Software, Inc., Syracuse, USA). This software enabled the correlation of the antenna configuration with specific laminate characteristics and conductance specifications to a specific frequency band. We optimized the antenna for a match frequency of *f* = 7.9 GHz. Simulation was parameterized for air, assuming a sample thickness of *d* = 0.76 mm and dielectrically properties with a relative permittivity of *ε*_*r*_ = 3.48 As/Vm of the laminate RO4350 for a patch antenna with one feed for the electromagnetic waves. To optimize for a specific frequency, the antenna configuration was calculated by the program and simulations of the emission pattern of the antenna were conducted.

**Fig 1:**
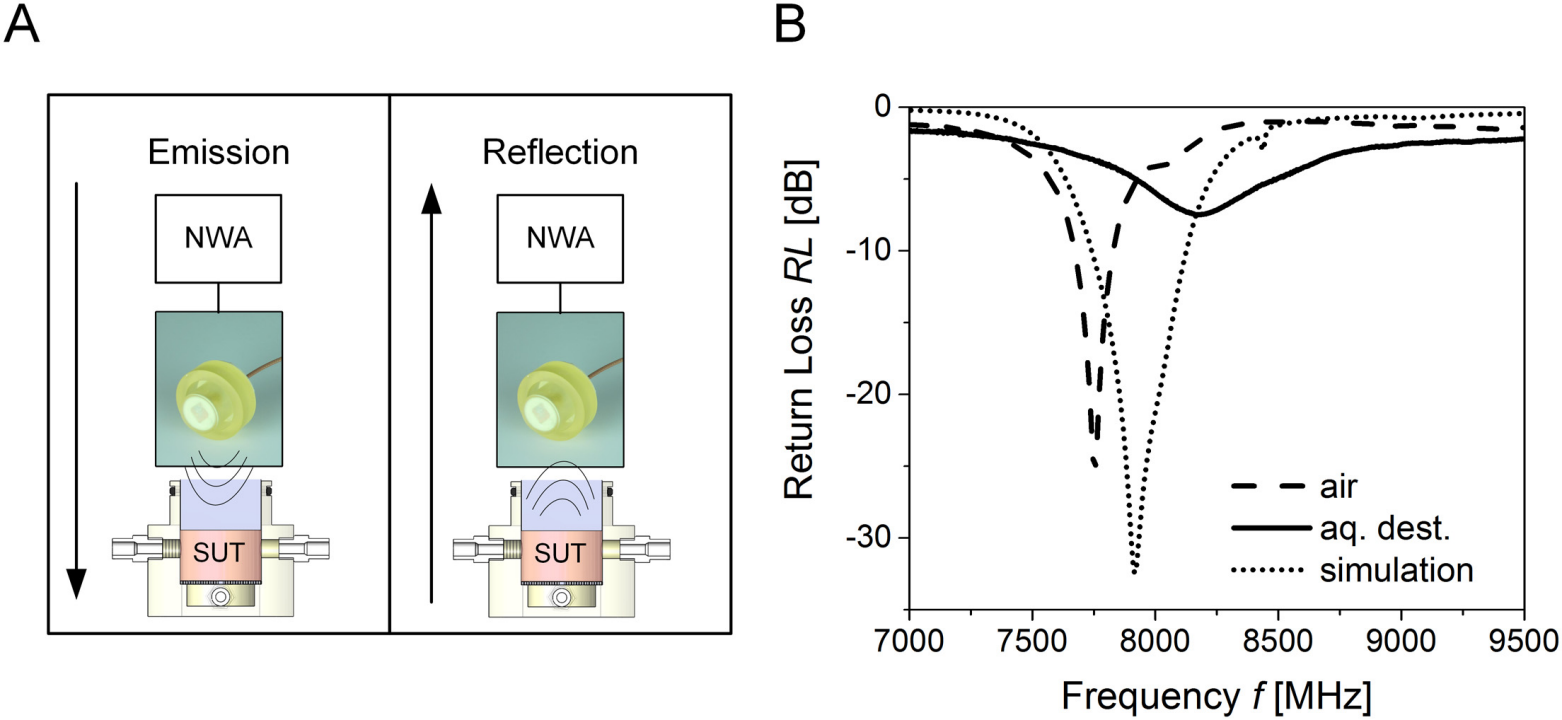
Microwave reflection measurement. (A) A patch antenna, optimized at *f* = 7.9 GHz, was connected to a network analyzer (NWA) and microwaves were introduced to a substance under test (SUT). Following microwave emission, the reflected signal was detected. (B) Each SUT exhibited a specific return loss (*RL*) and frequency at the local minimum (*f*_*min*_), exemplarily shown for purified water (aq. dest.) and air. Measurements were compared to a mathematical electromagnetic antenna model. The reflection minimum and frequency for air shifted in a range between 7000–9000 MHz for simulation and measurements.

The identified and manufactured antenna was then connected to a network analyzer (NWA) N9918A FieldFox Microwave Analyzer, 26.5 GHz (Agilent Technologies, Santa Clara, USA) to emit microwaves of a specific frequency. Measurements were performed following insertion of a substance under test (SUT) inside the bioreactor chamber comprising the patch antenna (Fig 1A). The SUT was either a control fluid or an *in vitro* model exhibiting consistent volumes. After interacting with the molecules of the sample, reflection signal spectra of microwaves were recorded by the NWA at a frequency range between 7.0 to 9.5 GHz. To evaluate the measured spectra, the amplitude of the return loss (*RL*) and the corresponding frequency (*f*_*min*_) were detected (Fig 1B). The *RL* was defined as the quotient of reflected and emitted power in decibel. The lower *RL*, the more signal is lost. The detection time per measurement including the analysis of 3000 data points was less than 1 second. Comparison of simulated and measured reflection spectra validated the computational-modeling-based optimization of the antenna.

### Sensitivity analysis of the measuring setup

Microwave reflection measurements with different alcohols (methanol, ethanol, and propanol) and sodium chloride (NaCl) at concentrations of 0.1, 10, and 20% were performed to assess the antenna sensitivity regarding osmolality and polarity. For these measurements, the bioreactor was occupied with the respective solutions with a total volume of *V* = 3.6 ml during the measurement.

### Cell isolation and culture

To isolate human epidermal keratinocytes (hEK) and human dermal fibroblasts (hDF), foreskin biopsies were washed with phosphate buffered saline without calcium and magnesium (PBS), cut into pieces of approximately 5 mm^2^ and treated with dispase (2 U/mL) at 4°C for 15 h. Following mechanical removal, the epidermis was incubated in 0.05% Trypsin/Ethylenediaminetetraacetic acid (EDTA) buffer at 37°C for 5 min. The enzymatic reaction was halted by adding 10% fetal calf serum (FCS) and a hEK suspension was obtained by disrupting the digested epidermal fragments by re-suspension. After centrifugation at 270-fold gravity for 5 min, the cell pellet was gently re-suspended in EpiLife^®^ medium supplemented with Human Keratinocyte Growth Supplements and 1% penicillin/ streptomycin, and cultured in a humidified incubator at 5% CO_2_ and 37°C. To isolate hDF, dermal fragments were cultured using Dulbecco’s Modified Eagle’s Medium (DMEM) supplemented with 10% FCS and 1% penicillin/ streptomycin in analogy to hEK culture.

### Generation of dermal and full-thickness skin equivalents

All *in vitro* models were based on a collagen type I hydrogel. For cell-free dermal equivalents, gel neutralization liquid (322.5 ml 2xDMEM; 7.5 ml 3 M 4-(2-hydroxyethyl)-1-piperazineethanesulfonic acid (HEPES); 1.25 ml chondroitin-4-sulfate; 1.25 ml chondroitin-6-sulfate; 7.5 ml FCS was mixed at a ratio of 1:3 with collagen type I exhibiting a concentration of *c* = 10 mg/ml in 0.1% acetic acid to a final total collagen mass of 24 mg (*c* = 6.67 mg/ml), 42 mg (*c* = 11.67 mg/ml), 60 mg (*c* = 16.67 mg/ml) or 96 mg (*c* = 26.67 mg/ml). After gelation, models were compressed as described.

Dermal equivalents were generated using 6-well ThinCert^™^ cell culture inserts and ThinCert^™^ plates (Greiner Bio-One GmbH, Frickenhausen, Germany). For the dermal component, either 4.5x10^5^ or 9.0x10^9^ fibroblasts per insert were suspended in gel neutralization liquid (322.5 ml 2xDMEM; 7.5 ml 3 M HEPES; 1.25 ml chondroitin-4-sulfate; 1.25 ml chondroitin-6-sulfate; 7.5 ml FCS) and mixed at a ratio of 1:3 with 42 mg collagen type I to a final volume of *V* = 7 ml. After gelation, models were compressed as described. Dermal equivalents were cultured under submerged conditions in DMEM/4.5 g/l glucose/1% L-glutamine/10% FCS/L-pyruvate at 37°C for 14 days. Cell culture medium was changed on a daily basis.

An epidermal component (human epidermis, rHE) was reconstructed to obtain full-thickness 3D skin models (FT 3D skin). Therefore, dermal equivalents seeded with 4.5x10^9^ fibroblasts were transferred to deep ThinCert^™^ plates (Greiner Bio-One GmbH) one day after generation and equilibrated with epidermal growth medium/ 10% FCS/ 1% penicillin- streptomycin/ 10mg/ml gentamycine for 2 h at 37°C. Following, 2x10^6^ hEK in Epilife^®^ medium were seeded on top of the dermal equivalent and incubated for 1.5 h at 37°C. Subsequently, skin equivalents were covered with Epilife^®^ supplemented with calcium chloride at 0.03 mM (Sigma Aldrich, St. Louis, USA). After 24 h, the cell culture medium was partially aspirated and air lift culture was initiated. Additionally, Epilife^®^ medium was supplemented with keratinocyte growth factor (Merck KGaA, Darmstadt, Germany) and ascorbate-2-phosphate (Sigma Aldrich, St. Louis, USA) and 73 mg/ml. Tissue constructs were cultured at the air liquid interface for 21 days to allow *stratum corneum* formation. Cell culture medium was changed on a daily basis.

### Skin equivalent preparation

To alter the osmolality of skin equivalents, tissues were incubated for 6 h either in sodium chloride at 20% or 0.1% (Carl Roth GmbH & Co.KG, Karlsruhe, Germany), mimicking severe (final osmolality after incubation 3700 mOsm/l) and moderate (final osmolality after incubation 315 mOsm/l) hypernatraemia [24,25], or in purified water (aq. dest.), mimicking hyponatraemia with a osmolality of 150 mOsml/l [26]. PBS incubation resulted in the physiological osmolality of 300 mOsml/l. Depending on the experimental setup, the epidermal layer of a full-thickness skin equivalent was removed, using forceps if required, to compare skin models with different components.

### Dry to wet weight measurement

The wet weight of the substance under test was determined following microwave reflection measurement by using a scale (Dietrich-Waagenbau Merkenbach GmbH, Herborn-Merkenbach, Germany). Then, samples were vacuum freeze dried for 48 h in a Christ Alpha 1–2 LD freeze drying device (Martin Christ Gefriertrocknungsanlagen GmbH, Osterrode am Harz, Germany) and wet to dry weight ratio was determined.

### Histological staining

To analyze the morphological characteristics, tissue samples were fixed using the paraformaldehyde based fixation agent Roti^®^-Histofix 4% (Carl Roth GmbH + Co. KG) and embedded in paraffin. From the paraffin-embedded samples, tissue sections with a thickness of 5 μm were obtained using a microtome (Microm HM 340E; Thermo Fisher Scientific; Waltham, USA). Following deparaffinization, sections were hematoxylin and eosin (H&E) stained. Bright-field images at a 20- or 40-fold magnification were acquired using a KEYENCE BZ-9000 microscope (Keyence Deutschland GmbH, Neu-Isenburg, Germany).

### Statistical analysis

Each data point comprised three individual samples that were measured in triplicate under equal conditions. Quantitative data were analyzed for statistically significant differences using a One-Way ANOVA employing the Dunnett’s significance test. For all statistical tests a *p*-value < 0.05 was considered as significant.

## Results

### Fluid osmolality and polarity result in characteristic reflection spectra

**Fig 2:**
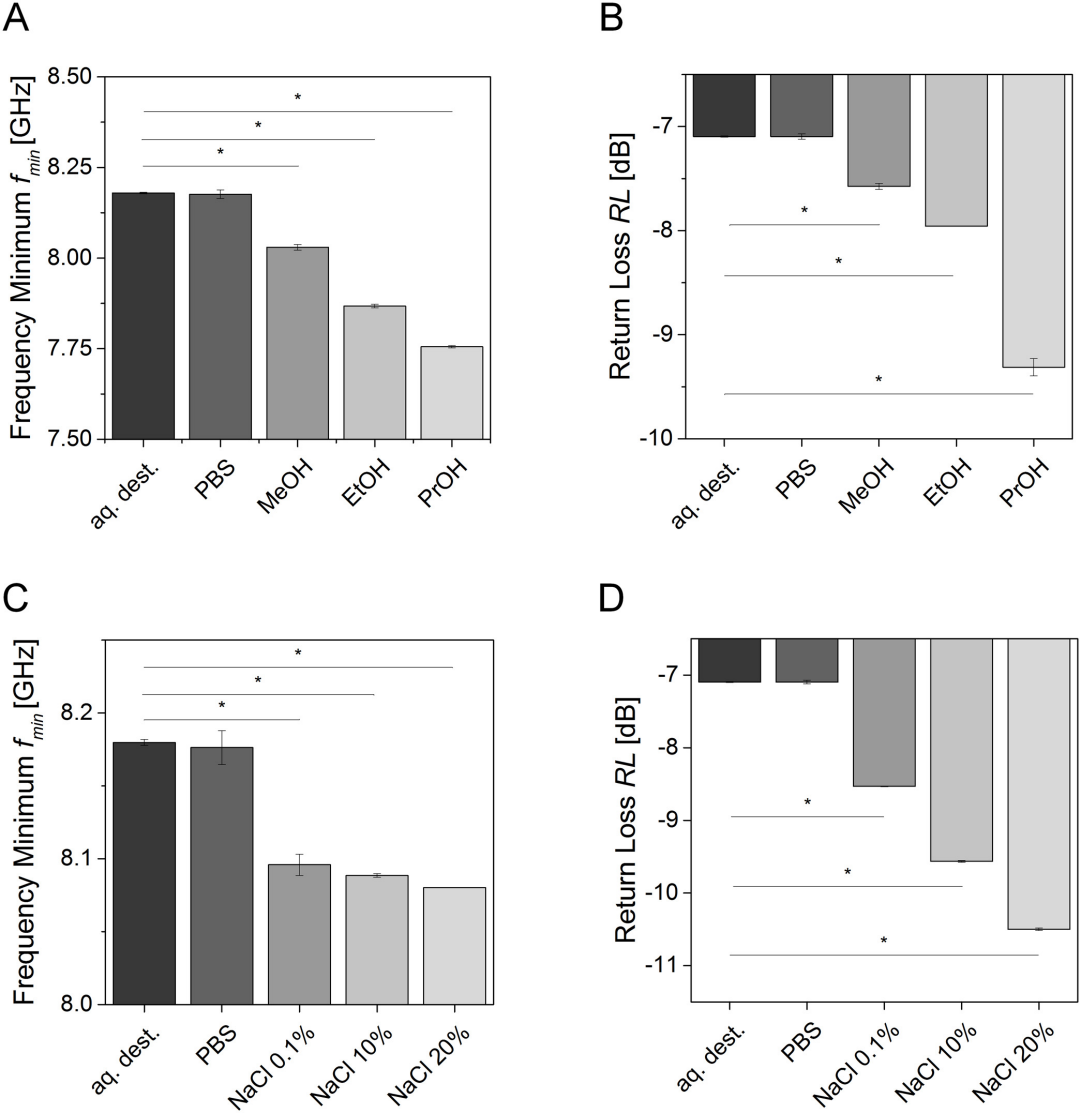
*Retun loss (RL)* and *f*_*min*_ of fluids exhibiting different polarities and osmolalities in comparison to purified water (aq. dest.) and phosphate buffered saline (PBS). (A) *f*_*min*_ decreased with lower polarity. Water; exhibiting the highest polarity showed the highest *f*_*min*_ compared to methanol (MeOH), ethanol (EtOH) and propanol (PrOH) with decreasing polarities. (B) *RL* decreased with lower polarity from aq. dest. to PrOH. (C) *f*_*min*_ decreased with higher osmolality from aq. dest. to sodium chloride (NaCl) solutions at concentrations ranging from 0.1 to 20%. (D) *RL* decreased with a higher osmolality. Bar diagrams show mean values and error bars depict standard deviation. Asterisks indicate statistical relevant differences (*p*-value ≤ 0.5). Each group comprised three individual samples that were measured in triplicate.

To characterize the sensitivity of the experimental setup, reflection spectra of fluids with different polarities and osmolalities were measured, and the *RL* and *f*_*min*_ at the local minimum were derived (Fig 2). Therefore, the effect of fluids with a declining polarity compared to purified water (aq. dest.) was investigated. The results demonstrated decreasing *f*_*min*_ with lower polarity such as for propanol (PrOH) with a mean value of *f*_*PrOH*_ = 7.75 GHz compared to aq. dest. and PBS with a mean value of *f*_*aq*.*dest*._ = 8.2 GHz (Fig 2A). The *RL* of the reflection signal detected by the NWA decreased with a lower polarity of the fluid (Fig 2B). The *RL* declined from a mean value of *RL*_aq.dest._ = -7.1 dB for aq. dest. to a mean value of *RL*_*ProOH*_ = -9.4 dB for PrOH demonstrating a significant influence of the polarity.

Since a constant osmolality of the fluids in the body is essential for well-being of tissues, we investigated the effect of different osmolalities using altered NaCl concentrations compared to aq. dest. and PBS. The results demonstrated declining values for *f*_*min*_ with a higher osmolality from an average of *f*_aq.dest._ = 8.18 GHz for aq. dest. to a mean value of *f*_*NaCl20%*_ = 8.09 GHz for 20% NaCl solution (Fig 2C). Additionally, the *RL* decreased with higher osmolality and shifted from an average value of *RL*_*aq*.*dest*._ = -7.1 dB for aq. dest. to a mean value of *RL*_*NaCl20%*_ = -10.5 dB for 20% NaCl (Fig 2D).

### Microwave-material interaction

To estimate sample heating during measurement, we calculated the expected temperature increase of the SUT. Due to a power of *P* = 0.631 mW generated by the NWA and a measurement time of 1 second, the total energy input *Q* into the sample was 0.631x10^-3^ J. Based on this energy input, the temperature increase can be calculated according to the definition of the specific heat capacity solved for the temperature difference *ΔT* [K]

$$\Delta T=\frac{Q}{c\;m}$$ (1)

Herby, Eq 1 was parameterized for water that represented approximately 95% of the total sample. For a specific heat capacity of *c* = 4.19 kg/kJ K and a mass of *m* = 3.6 g, the maximum heating induced by the microwaves under measurement conditions was calculated to *ΔT* = 4.183x10^-6^ K.

In order to investigate how deep microwaves penetrate into the SUT at an emission frequency of *f* = 8.2 GHz, we calculated the penetration depth *δ* [m]. The penetration depth was defined as the distance from the surface exhibiting 37% of the electric field strength compared to the initial field strength. Thereby, *δ* also considers the skin-effect of electromagnetic waves [27]. The penetration depth further depends on the circular frequency *ω* [Hz], the permeability *μ* [N/A^2^] and the electrical conductivity *σ* [S/m]:

$$\delta =\sqrt{\frac{1}{\omega \;\mu \;\sigma }}$$ (2)

In analogy to Eq 2, the penetration depth into the sample was *δ* = 1.758 cm. Thus, the whole sample exhibiting a height of 0.8 cm was exposed to the electromagnetic field and the reflected signal constituted a parameter that was also representative for deeper parts of the sample.

### Microwave technology allows characterizing the hydration of cell-free dermal equivalents

**Fig 3:**
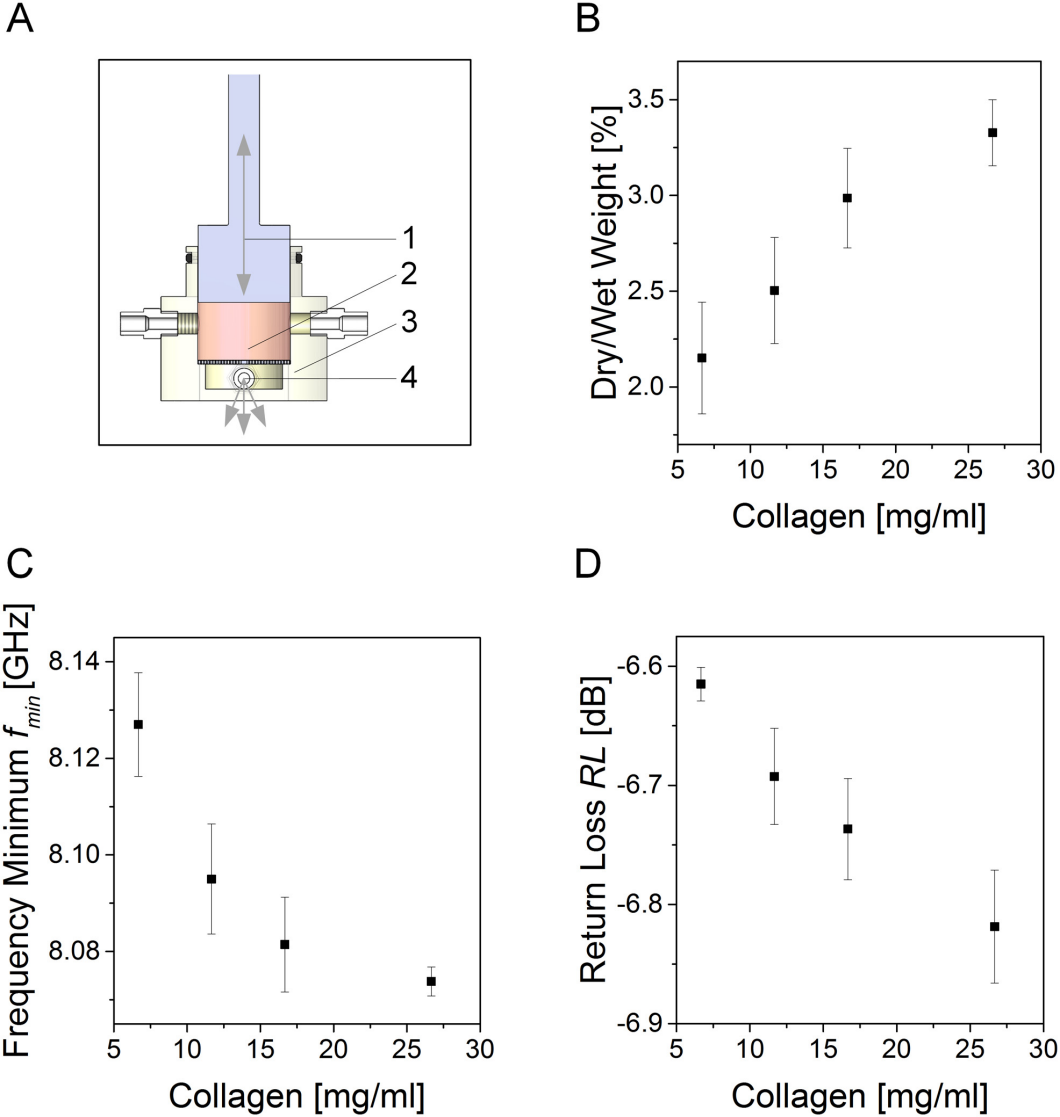
Measurement of cell-free dermal equivalents at different collagen concentrations. (A) A plastic compression system allowed generating dermal equivalents exhibiting collagen concentrations ranging from *c* = 6.67 mg/ml to *c* = 26.67 mg/ml. During processing, hydrogels were compressed to a defined volume of *V* = 3.6 cm^2^ employing a linear motion engine. The squeezer (1) compressed the hydrogel (2) in the bioreactor (3). Pried water was aspirated via the port near the bottom of the chamber (4). (B) Comparison of dry to wet weight showed an increase in weight with a higher collagen concentration, whereas (C) *f*_*min*_ at the detected minimum declined with a higher collagen concentration, and (D) *RL* decreased at higher collagen concentration. Plots show mean values, error bars depict standard deviation. Each group comprised 3 individual samples that were measured in triplicate.

To provide more complex and biologically-relevant test scenarios compared to the control fluids, cell-free dermal equivalents exhibiting a defined salt and water concentration were generated. Plastic compression was performed as described, allowing a final volume of the dermal equivalents of *V* = 3.6 cm^3^ (Fig 3A) to ensure standardized experimental conditions. The quantification of collagen concentration and comparison of dry and wet weight confirmed declining water contents at higher collagen concentrations (Fig 3B). With increasing collagen concentrations in the hydrogel, *f*_*min*_ decreased from a mean value of *f*_*24mg*_ = 8.125 GHz for hydrogels containing 24 mg collagen (*c* = 6.67 mg/ml) to an average frequency of *f*_*96mg*_ = 8.075 GHz for the samples containing 96 mg collagen (*c* = 26.67 mg/ml) (Fig 3C). Concurrently, *RL* was lowest for the highest collagen concentration, decreasing from a mean value of *RL* = -6.61 dB for 24 mg collagen to an average *RL* = -6.82 dB for 96 mg collagen per dermal equivalent (Fig 3D).

### Non-destructive hydration characterization of dermal equivalents using microwave technology

**Fig 4:**
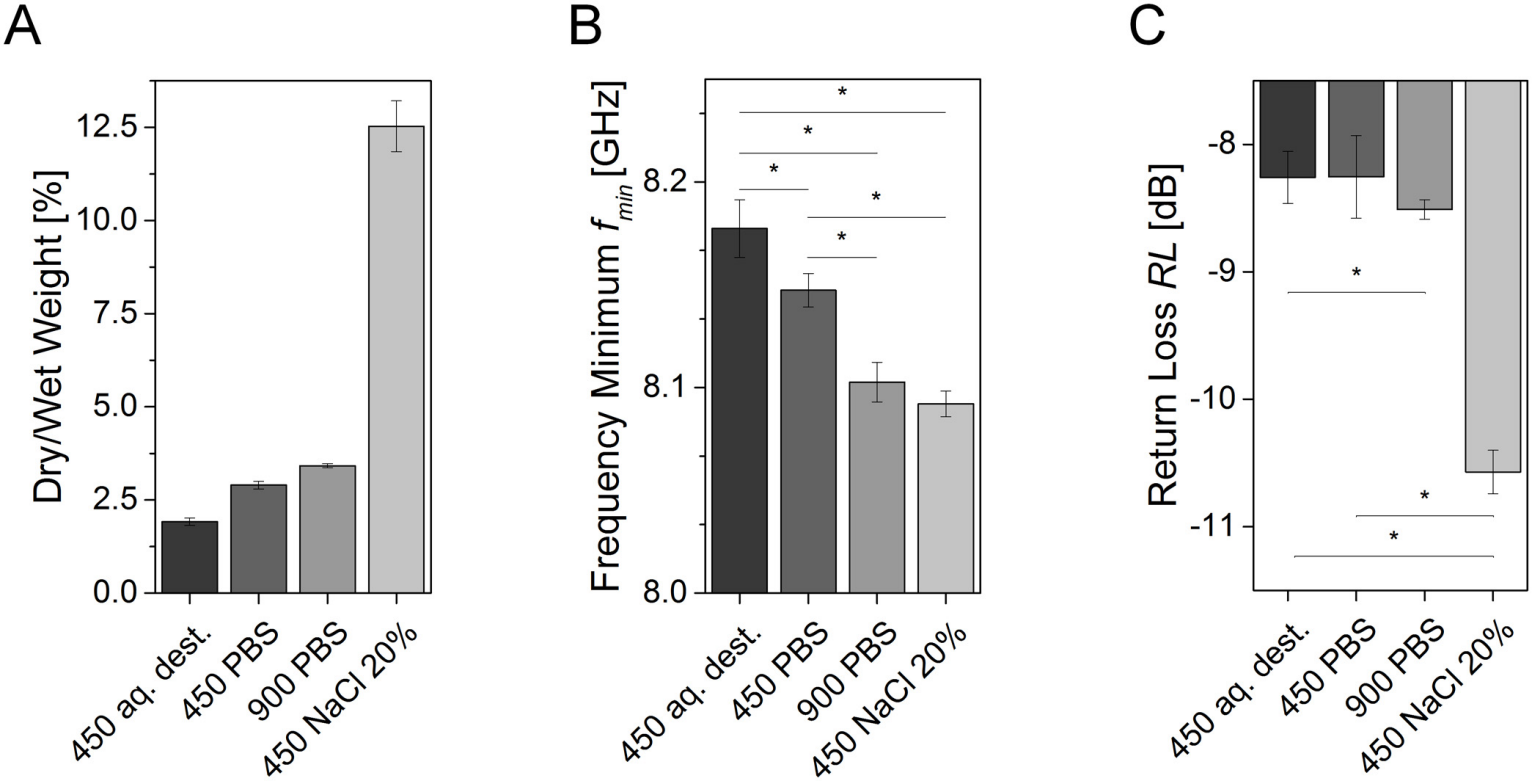
Microwave reflection signal of dermal equivalents at different osmolalities and cell concentrations. (A) Comparison of dry to wet weight ratio demonstrated that the weight of the dry component increased with a higher osmolality and cell concentration of the remaining substances. (B) *f*_*min*_ declined at higher cell concentrations (from 4.5x10^5^ to 9x10^5^ cells) and osmolalities, mimicking hypo-, iso- and hypernatraemia. (C) In analogy to *f*_*min*_, *RL* dropped with a higher osmolality. Bar diagrams show mean values, error bars depict standard deviation. Asterisks indicate statistical relevant differences (*p*-value ≤ 0.5). Each group comprised 3 individual samples that were measured in triplicate.

Fig 4 depicts the effect of cells and different salt concentrations on the microwave signal reflection of collagen type I dermal equivalents at different hydration scenarios. Assessing the dry to wet weight ratio, NaCl-treated models revealed the highest dry weight caused by deposition of salt molecules (Fig 4A). Salt-saturated hydrogels contained additional NaCl ions and dry weight was increased. Regarding *f*_*min*_, two effects were detected (Fig 4B). *f*_*min*_ decreased with higher osmolalities from aq.-dest.-treated (hyponatraemia) dermal equivalents (*f*_*450aq*.*dest*._ = 8.175 GHz) to *f*_*450PBS*_ = 8.16 GHz for the PBS-treated samples (isonatraemia) down to an average frequency of *f*_*450 NaCl20%*_ = 8.09 GHz for NaCl-treated models (hypernatraemia). Moreover, *f*_*min*_ seemed to be dependent on the cell density as well. At a constant osmolality, models containing 4.5x10^5^ hDF (abbreviation 450) per gel showed a higher *f*_*min*_ compared to the models seeded with 9x10^5^ cells (abbreviation 900). Here, a mean value of *f*_*900PBS*_ = 8.10 GHz was measured for *f*_*min*_.

Considering *RL*, values decreased from consistent amplitude of *RL*_*450PBS*, *aq*.*dest*._ ≈ -8.3 dB for PBS and aq. dest. samples to *RL*_*NaCl20%*_ = -10.7 dB for the models treated with NaCl (Fig 4C). Additionally, a minor effect of a changing cell density to the *RL* was detected, nevertheless, the impact of osmolality was higher.

### Characterization of full-thickness skin equivalents and the effects of the epidermal layer to microwave-based hydration quantification at the air-liquid interface

**Fig 5:**
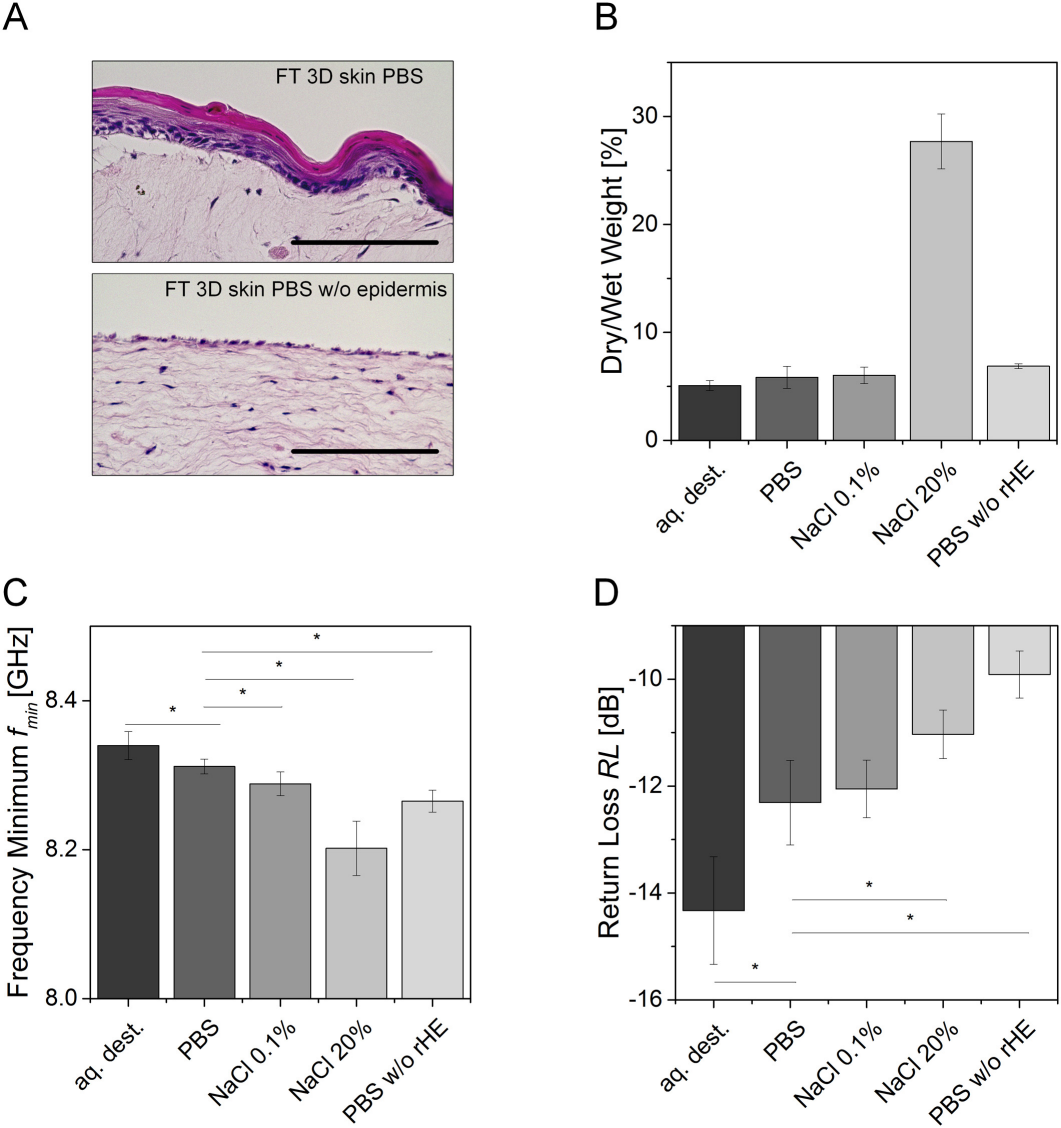
Microwave signals of 3D skin models at different osmolalities. (A) H&E-staining of full-thickness 3D skin models (FT 3D skin) with intact and removed epidermal layer following treatment with PBS. (B) The dry to wet weight ratio increased with higher osmolality (from aq. dest. to NaCl), whereas (C) *f*_*min*_ decreased with a higher osmolality. (D) *RL* increased with a higher osmolality and was highest where no epidermal layer was present (PBS w/o rHE). All scale bars indicate 50 μm. Bar diagrams show mean values, error bars depict standard deviation. Asterisks indicate statistical relevant differences (*p*-value ≤ 0.5). Each group comprised 3 individual samples that were measured in triplicate.

Fig 5A depicts the comparison between FT 3D skin equivalents with intact and removed epidermal layer. The dry to wet weight measurement revealed no difference between equivalents with intact or removed epidermal layer, when incubated with PBS, 0.1% NaCl, or aq. dest. (Fig 5B). NaCl-treated skin models at a NaCl concentration of *c* = 20% with an epidermal layer showed the highest dry to wet weight ratio with an average value of approximately 27% compared to approx. 5 to 7% for all other conditions. As stated for the dermal models, the dry weight was influenced by salt deposition.

Considering *f*_*min*_, two major impacts were found. In contrast to osmolality that resulted in lower values for *f*_*min*_ at higher osmolality, the presence of an epidermal layer shifted *f*_*min*_ to higher frequencies (Fig 5C). Significant differences of *f*_*min*_ were detected between PBS-incubated (physiological osmolality) to all other conditions. Moreover, the previously identified tendency of *RL* to lower values with rising osmolalities for dermal equivalents was reversed (Fig 5D). *RL* rose with an increasing osmolality from a mean value of *RL*_*aq*.*dest*_. = -14.25 dB for aq. dest. to *RL*_*NaCl20%*_ = -11.0 dB for skin models incubated with NaCl at *c* = 20%. The highest *RL* was measured for models with removed epidermis with an average value of *RL*_*PBS w/o rHE*_ = -10.0 dB. Compared to PBS-incubated models with an intact epidermis (*RL*_*PBS*_ = -12 dB), a recovery of *RL* was observed, which demonstrated the impact of the epidermal layer on the *RL*.

### Microwave based measurements facilitates distinction between defined groups

**Fig 6:**
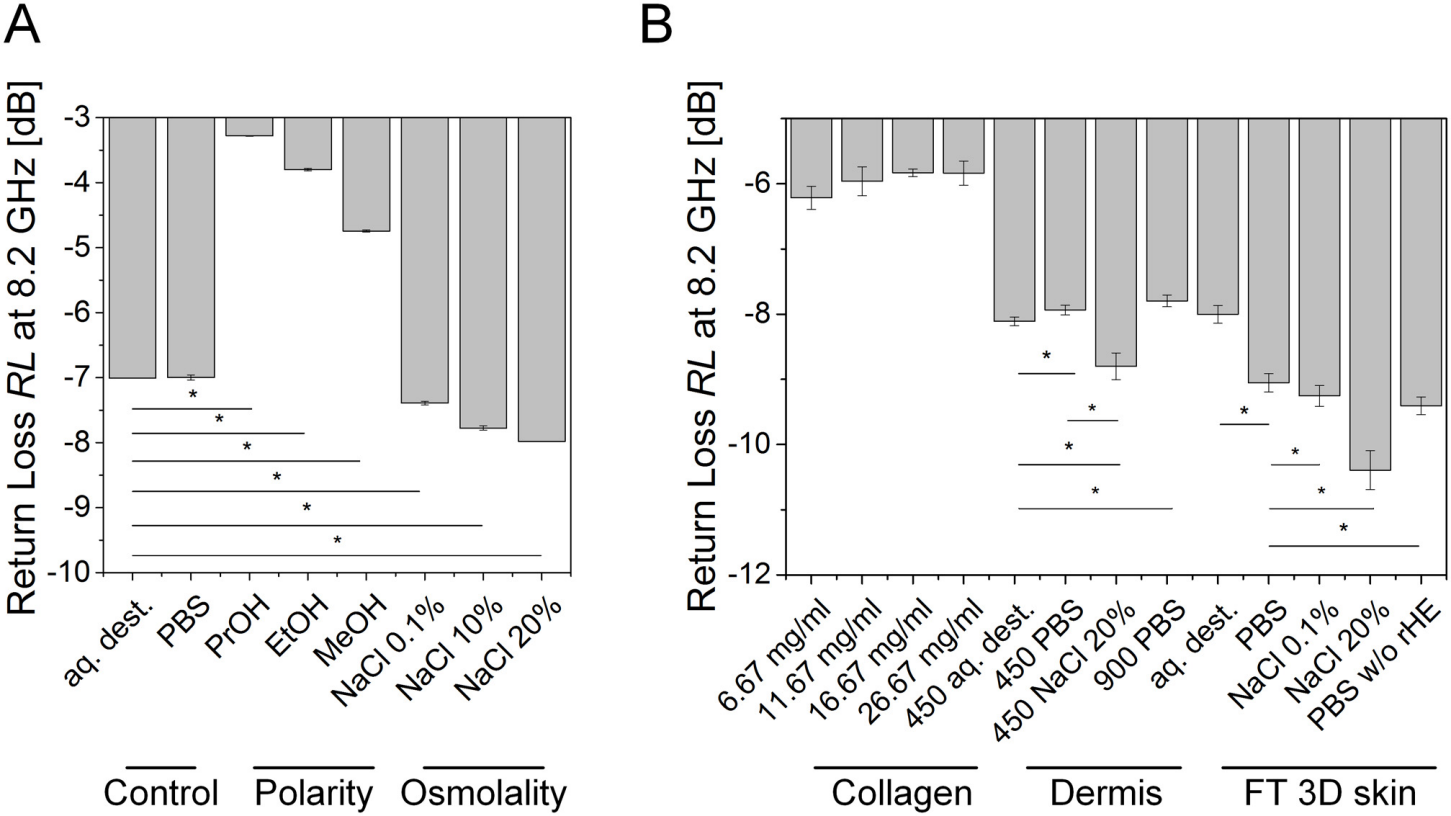
Microwave signal of fluids and *in vitro* test models measurements at 8.2 GHz. (A) Microwave measurements with fluids. Compared to Control, *RL* showed increased values for polarity measurements (Polarity) at a constant frequency of 8.2 GHz with a lower polarity from aq. dest. to PrOH. Osmolality measurements displayed a decreasing *RL* with the highest osmolality at NaCl 20%. (B) Microwave measurements with *in vitro* models. *RL* displayed increasing values at a constant frequency of 8.2 GHz for measurements with cell-free collagen hydrogels (Collagen). *RL* dropped with higher osmolalities for both dermal (Dermis) and full-thickness 3D skin models (FT 3D skin). Bar diagrams depict mean values, error bars depict standard deviations. Asterisks indicate statistical relevant differences (*p*-value ≤ 0.5). Each group comprised 3 individual samples that were measured in triplicate.

In order to investigate sensor robustness and to test whether processing costs for signal analysis can be reduced, all prior shown experiments were analyzed at a constant frequency of 8.2 GHz. Fig 6A depicts the results obtained from fluid sensitivity measurements. Compared to the controls aq. dest. and PBS, both at *RL* ≈ -7.00 dB, the values for the measurements of different polarities showed an increasing *RL* with lower polarity up to *RL*_*PrOH*_ = -3.28 dB for PrOH. In contrast, osmolality measurements revealed decreasing values for higher osmolalities from the controls to NaCl 20% with *RL*_*NaCl20%*_ = -7.98 dB.

Considering *RL* at a frequency of 8.2 GHz for all *in vitro* models (Fig 6B), cell-free collagen hydrogels showed slightly increasing values with a higher collagen concentration from *RL* = -6.22 dB for *c* = 6.67 mg/ml collagen up to *RL* = -5.83 dB for *c* = 26.67 mg/ml collagen. As already shown for fluids of changing osmolality, dermal and FT 3D skin models exhibited a decreasing *RL* with rising osmolality. For dermal equivalents, the values decreased from around -8 dB for aq. dest.- and PBS-treated models down to *RL*_*450NaCl*_ = -8.80 dB for NaCl-treated models. For FT 3D skin, the values decreased from *RL*_*aq*.*dest*._ = -8.00 dB for aq.-dest.-treated samples down to *RL*_*NaCl20%*_ = -10.39 dB for NaCl-treated samples at a NaCl concentration of *c* = 20%. *RL* was 0.35 dB lower for the PBS-treated samples with removed epidermal layer than with epidermal layer, confirming the impact of the epidermal layer of the skin models. Considering osmolality changes in FT 3D skin, significant differences between physiological conditions (PBS) and a moderate hypernatraemia (0.1% NaCl) could be detected.

## Discussion

Since water-related diseases cause more than 5 million deaths per year [2], hydration represents a crucial vital parameter especially for elderly people, in intensive care, or during hard laborious work and training. Thus, hydration should be monitored preventively for instance via PHM. A PHM method must compete with high standards for medical devices according to 93/42/EWG and needs to be approved within a conformity assessment procedure to ensure safety, robustness and functionality. Additionally, PHM systems should support non-invasive, fast, specific, and reliable measurements. To facilitate applicability in everyday life and special care, a PHM method further requires system integration into a small wearable device. Microwave-based measurement of hydration at the skin surface basically claims to fulfil those requirements. In order to investigate whether a microwave-based sensor can be employed to monitor relative changes of tissue hydration state, a sensor for reflection measurements at microwave frequencies was developed.

Monitoring hydration through the skin is currently based on invasive measurements [28]. Thus, skin barrier is affected entailing limitations such a risk of contamination. An alternative method to measuring hydration at the skin is to sample and analyze salvia. It has been shown, that salvia composition allows estimating hydration state [29,30]. Nevertheless, a microwave-based hydration sensor technology bears several advantages in comparison to a salvia-based sensor. For instance, the microwave sensor antenna is manufactured of economic components. Additionally, no disposable components are required and the sensor itself is time-independent. No shift of the measured values or the properties of the sensor over time are expected due to usage or ageing. Furthermore, the microwave sensor allows continuous non-invasive measurement. The antenna can be placed as patch on the skin. Continuous measurement of hydration via composition of saliva seems to be difficult since salvia needs to be sampled. For the microwave-based sensor, no sampling is required. Thus, no manual operations are necessary.

The sensor antenna demonstrated a sufficient sensitivity allowing distinguishing between osmolalities and polarities of pure control liquids when using the maximum *RL* at varying *f*_*min*_ or the local *RL* at a constant frequency of 8.2 GHz as readout. In addition, the collagen concentration, and thus the water content in the hydrogel could be detected in cell-free dermal models employing the maximum *RL* under constant osmolality. During the measurements, collagen concentrations covered a broad range of collagen concentrations found in human adults [31], which supports a rapid transfer of the technology for *in vivo* usage. Furthermore, water loss, which was induced by varying collagen concentrations, resolved several levels of minimal dehydration that is characterized by a water loss of less than 3% [32]. The sensor allowed distinguishing between these small changes of water content in the tissues. For dermal models containing hDF, hyponatraemia, severe hypernatraemia, and normhydration were mimicked. Here, salt concentrations covered pathological salt depletion [26], physiological salt concentration, and high salt accumulation in skin [24,25]. Incubation in PBS resulted in an osmolality of 300 mOsml/l that is considered as physiological. All osmolality conditions, could be differentiated using the maximum *RL* at varying *f*_*min*_ or the local *RL* at a constant frequency as measuring readout. Interestingly, hDF-induced remodeling and contraction of the dermal models were detectable in the measurements. Herby, *f*_*min*_ shifted during culture when comparing cell-free and cell-seeded hydrogels. Hydrogel remodeling was also indicated by varying dry to wet weight ratios. A significant shift of *f*_*min*_ during culture was found for both cell concentrations. This underlines a high sensor sensitivity. The feasibility of the sensor was confirmed, when increasing the complexity of the skin models by introducing an epidermal layer onto the cell-seeded dermal models. For FT 3D skin, a small osmolality increase was tested in addition to severe salt accumulation. Incubation in 0.1% NaCL solution, resulted in an osmolality of 315 mOsml/l that is considered as moderate hypernatraemia. *RL* as well as *f*_*min*_ turned out to be suitable readouts to detect the two pathological hydration states pathological salt depletion (aq. dest.) and severe salt accumulation (NaCl 20%). Moreover, moderate hypernatraemia (0.1% NaCl) resulted in a significant difference of *f*_*min*_ compared to physiological osmolality (PBS). By analyzing the measurements at a constant frequency *f* = 8.2 GHz—the maximum *RL* of aq. dest.–sensitivity obtained at different frequencies was reproduced. For cell-free and cell-seeded dermal models, measurements exhibited low variances as well as a high reproducibility within a group. Thus, it is also possible to distinguish between different hydration states and system complexities at a constant detection frequency. Regarding implementation of microwave measurements for PHM, analysis will require less computational cost and the complexity of the electronic components can be reduced.

Interestingly, the microwave-based sensor also supported the discrimination between different skin layers. The presence of an epidermal layer resulted in a significant impact on the reflected signal for varying and constant detection frequencies. For FT 3D skin models, differences between *RL* and *f*_*min*_ of intact and removed epidermis can be attributed to the surface humidity of the SUT. In contrast to the dermal models, epidermal models exhibited a dry surface. Additionally, the epidermis comprises the highly-structured *stratum corneum*, inducing further signal reflections. Furthermore, as already shown for impedance analysis, the epidermal layer has capacitive effects, and parts of the emitted energy are absorbed and less signal is reflected [33]. When removing the epidermal layer, reflected signal tended towards the signal of dermal models. This fact demonstrates the interaction between electromagnetic waves and distinct skin components. Thus, the technology is not limited to material and fluid analysis, but also bears potential to characterize complex biological tissues properties such as skin integrity.

The experimental setup was based on components supporting miniaturization as a prerequisite for the integration into a wearable or patch device that can be placed on healthy skin. Additionally, the required power of the high frequency microwaves used in the measurements was kept within the range defined by the international commission on radiological protection (ICRP) for tissue reactions as well as the valid electromagnetic compatibility guideline DIN EN 60601 for medical devices [34,35]. With a power output of *P* = 0.631 mW emitted by the NWA, no relevant temperature increase is entailed, and thus no adverse effects, such as degeneration, or loss of viability of the skin models are expected. Due to the calculated penetration depth, microwaves were introduced into the whole sample; facilitating investigation of dermal hydration state also in deeper tissue regions. An additional advantage of the system is the short detection time per measurement of less than 1 second even if a wide frequency range and many data points are analyzed. Optimized for usage as a medical device, only the most relevant frequencies, such as shown in this study at *f* = 8.2 GHz, can serve as readout for an even better data acquisition time.

To translate the sensor into a PHM system, several uncertainties must be addressed. In our study, the FT 3D skin provided a reproducible *in vitro* test system for sensor development and characterization. Such skin equivalents represent a reliable tool in chemical and pharmacological research [22,23]. However, FT 3D skin does only partially resemble the complexity of native skin. Moreover, some physiological functions of native skin are missing, and untested variables could impact the reflection spectra, when measuring *in vivo*. For example, blood vessels were absent and due to the lack of skin appendages, no perspiration occurred. Furthermore, concentration variations in the interstitial fluid exhibit a low dynamic in skin equivalents. These parameters and conditions could alter the sensor signal. Additionally, FT 3D skin represented a healthy tissue and the impact of pathological states on measured spectra could not be investigated *in vitro*. When interpreting results obtained using skin equivalents, these limitations should be considered. A potential approach to address these uncertainties is to embed the microwave sensor into a multi sensor array. The biological variance of skin, which was also partially detected in the presented study, could then be determined and compensated by complementary non-invasive technologies, when measuring *in vivo*. Studies employing impedance spectroscopy for the measurement of the trans-epidermal barrier [33] as well as the characterization of the osmolality state or the perspiration rate of a tissue [12,36] are promising candidates for the optimization of the information content on the sample. Such an approach could ensure systems robustness and facilitate handling of a high number of degrees of freedom. In our study, the number of degrees of freedom was artificially controlled by varying only one parameter per measurement. Since the sensor measures only relative changes of tissue hydration, it is additionally required to individualize the sensor via adaptive calibration algorithms. The combination of established non-destructive monitoring methods and sophisticated data analysis technologies with the microwave system could help to generate a clear picture of the skin constitution *in vitro* and *in vivo* non-invasively.

## Conclusion

By now, microwave-based analyses are focused on material and liquid characterization [17,18,37,38]. The technology has only partially been employed for the characterization of biological tissue *in vivo* or *in vitro* such as microwave tomography [39,40]. Our study demonstrates the basic feasibility of microwave sensor technologies to measure relative hydration changes at the interface between biological tissue and micro-electronics. The system can be used for the non-destructive characterization of relative hydration or integrity of *in vitro* skin equivalents, e.g. as quality criteria. The *in vitro* characterization of the sensor indicates that microwaves could serve as an integral part of a medical device for hydration monitoring. Nevertheless, for a final proof of feasibility, a clinical study must be performed.
